# Shared Activities as a Protective Factor Against Behavioral and Psychological Symptoms of Dementia and Caregiver Stress

**DOI:** 10.1093/geroni/igae034

**Published:** 2024-03-11

**Authors:** Darina V Petrovsky, Mustafa Yildiz, Maria Yefimova, Justine S Sefcik, Zachary G Baker, Kris Pui Kwan Ma, Zahra Rahemi, Juanita-Dawne R Bacsu, Matthew Lee Smith, Carolyn E Z Pickering

**Affiliations:** Division of Women, Children, & Families, Duke University School of Nursing, Durham, North Carolina, USA; Department of Research, Jane and Robert Cizik School of Nursing, University of Texas Health Science Center at Houston, Houston, Texas, USA; Center for Nursing Excellence & Innovation, University of California, San Francisco, San Francisco, California, USA; Department of Physiological Nursing, UCSF School of Nursing, San Francisco, California, USA; College of Nursing and Health Professions, Drexel University, Philadelphia, Pennsylvania, USA; Edson College of Nursing and Health Innovation, Arizona State University, Phoenix, Arizona, USA; Department of Family Medicine, University of Washington, Seattle, Washington, USA; School of Nursing, Clemson University, Greenville, South Carolina, USA; School of Nursing, Thompson Rivers University, Kamloops, British Columbia, Canada; Department of Health Behavior, Texas A&M University School of Public Health, College Station, Texas, USA; Department of Research, Jane and Robert Cizik School of Nursing, University of Texas Health Science Center at Houston, Houston, Texas, USA

**Keywords:** Activities, Caregiving, Dementia, Quantitative research methods, Stress

## Abstract

**Background and Objectives:**

Most persons with dementia experience behavioral and psychological symptoms (BPSD). While there is evidence that structured activity programs can be beneficial for persons with dementia and their caregivers, it is not well understood how joint engagement in shared activities affects BPSD and caregiver stress. The purpose of this study was to examine the moderating effect of doing a shared activity on the BPSD and caregiver stress.

**Research Design and Methods:**

This study used an intensive longitudinal observational design in which caregivers completed baseline and once-a-day diary surveys for 21 days. Caregivers were asked whether they did a pleasant noncare activity with their relative, the presence of 8 BPSD, and their stress level. A moderation model in a structural equation model examined the relationship between these variables.

**Results:**

Our sample consisted of 453 caregivers (87.4% female, 51.4% non-Hispanic White, mean age 53 years [standard deviation {*SD*}: 14]) and person living with dementia whose mean age was 79 years (*SD*: 9). On days when the caregivers engaged in a shared activity together with person living with dementia, there was a significant decrease in the BPSD (estimate −0.038, standard error [*SE*] = 0.016, 95% confidence interval [CI]: −0.069, −0.007, *p* value = 0.018). The effects of engaging in a shared activity decreased the impact of caregiver stress by 0.052 (estimate −0.052, *SE* = 0.018, 95% CI: −0.087, −0.017, *p* value = 0.004). At the between-person level, no differences were found in BPSD across caregivers who engaged or did not engage in shared activities.

**Discussion and Implications:**

The results of our study indicate that doing a shared activity is associated with reduced BPSD among persons with dementia and may buffer the impact of caregiver stress on BPSD. Shared activities should be considered a key intervention component for dementia caregivers.

**Translational Significance:** Behavioral and psychological symptoms (BPSD) in persons with dementia are difficult to manage at home. This intensive longitudinal study focuses on engaging in shared activities and its relationship to BPSD and caregiver stress. Our study findings indicate that engaging in shared activities is a protective factor against BPSD among community-dwelling caregivers and persons with dementia. Interventions that incorporate shared activities into daily life (e.g., listening to music during mealtimes) may be more effective and scalable than prescribed activities because these shared activities address the unmet needs of a person living with dementia while also helping caregivers manage their stress.

## Background and Objectives

Behavioral and psychological symptoms among persons living with dementia (BPSD), which include delusions, hallucinations, agitation, aggression, depression, anxiety, apathy, disinhibition, irritability, motor disturbances, and sleep disturbances, are distressing and pervasive (Kales et al., 2015). The prevalence of BPSD varies between 4% and 38% depending on the type of symptom being examined (Kwon & Lee, 2021; Leung et al., 2021). BPSD fluctuate over time and present a significant challenge for persons living with dementia and their caregivers at home. Moreover, developing effective therapies for BPSD is challenging due to the changing trajectories and the unpredictability of BPSD (Vik et al., 2018).

Older adults with BPSD are more likely to experience poor quality of life (Hodgson et al., 2014), longer hospital stays, and nursing home placement (Ferreira et al., 2023; Wulff et al., 2010). As a result of BPSD, caregivers encounter increased distress (Khoo et al., 2013), burden (Baharudin et al., 2019; Okura & Langa, 2011; Ryan et al., 2012), and burnout (Hiyoshi-Taniguchi et al., 2018). In the context of daily caregiving, the stress associated with managing ongoing BPSD significantly predicts abusive and neglectful behaviors among caregivers of persons living with dementia (Pickering et al., 2020). In a prior cross-sectional study with 250 persons living with dementia and caregiver dyads, increased caregiver burden and depression were associated with greater levels of harsh communication between the dyad members (Petrovsky et al., 2019). In a separate study of 256 caregivers and persons living with dementia, greater use of harsh communication was associated with increased caregiver burden and frustration with caregiving (Leggett et al., 2019).

According to the Progressively Lowered Stress Threshold Model (Hall & Buckwalter, 1987; Smith et al., 2004), older adults living with dementia accumulate stress throughout the day, but, due to their cognitive impairment, are unable to cope with multiple stimuli. Their stress threshold is reduced, resulting in BPSD, such as agitation, night awakenings, and combativeness (Hall & Buckwalter, 1987; Smith et al., 2004). Previous research has found that caregivers’ neglectful and aggressive behaviors can worsen BPSD. For example, persons living with dementia are more likely to exhibit BPSD on the same and the next day after experiencing neglectful, psychological, and physical aggressive behaviors from caregivers (Pickering et al., 2022). This suggests that caregivers’ behaviors may lower stress threshold of persons living with dementia, thus making them more likely to exhibit BPSD. Given persons living with dementia often interact with caregivers and that the dyadic interactions play an influential role in BPSD occurrence, it is important to identify factors in these caregiver–care recipient interactions that can mitigate and buffer against BPSD.

Emerging research suggests that shared activities between persons living with dementia and caregivers can have positive outcomes for each person in the dyad. For example, in the Tailored Activity Program (TAP), an occupational therapist assesses the abilities and interests of persons living with dementia and instructs caregivers in using prescribed tailored activities to decrease the number of BPSD. These activities hold meaning to persons living with dementia and are tailored according to the cognitive level. They include singing familiar songs, reminiscing about the past, playing games, or engaging in arts and crafts among others. As a result of TAP, caregivers experienced improved well-being and reported enhanced skills and less upset with BPSD (Gitlin et al., 2009, 2018, 2021). For persons living with dementia, a reduced number and frequency of BPSD as well as improved engagement and pleasure have been identified when using activities prescriptions (Gitlin et al., 2009). Likewise, caregivers who participated in the Resources for Enhancing Alzheimer’s Caregiver Health study, which included doing small, pleasant activities together along with learning other behavioral mood management skills experienced fewer depressive symptoms and greater use of adaptive coping strategies (Gallagher-Thompson et al., 2003).

While there is evidence that structured activity programs can be beneficial for dyads, it is not well understood how joint engagement in shared, pleasant activities that happen organically affect both the caregiver and the person living with dementia. In a study by Sideman and colleagues (2023), caregivers reported feeling connected with the person living with dementia when engaging in everyday activities, such as playing or making music together. However, more research is needed to understand what occurs between caregivers and person living with dementia and identify opportunities for shared activities incorporated into the daily routine, rather than setting aside time/resources for a prescribed activity. Identifying whether there is a decrease in both BPSD and caregiver stress could have implications on caregiving training content that teaches to regularly incorporate shared activities into daily routine. Therefore, the purpose of this study was to examine the moderating effect of doing a shared activity on the BPSD and caregiver stress. Our hypotheses were as follows.

1. *Hypothesis 1*: On a given day, when a caregiver engages in a shared activity, there will be a significant decrease in the number of BPSD.
2. *Hypothesis 2*: On a given day, doing a shared activity will decrease the impact of caregiver stress on BPSD.
3. *Hypothesis 3*: Across caregivers, on average, in cases where caregivers report engaging more often in shared activities, persons living with dementia will experience fewer BPSD compared to cases where caregivers report not engaging in a shared activity.

## Research Design and Methods

### Design

This study used an intensive longitudinal design in which caregivers completed baseline and once-a-day diary surveys for 21 days, responding to questions about their caregiving, environmental, and daily experiences.

### Participants

Participants consisted of a convenience sample of 453 community-dwelling caregivers for persons living with dementia who were 18 years or older. Caregivers provided unpaid care or assistance to the person living with dementia with mild cognitive impairment or dementia on at least two instrumental activities of daily living or one activity of daily living. Mild cognitive impairment or dementia was assessed using the Ascertain Dementia (AD8) interview (Galvin et al., 2006). To be included in the study, caregivers needed to reside with the person living with dementia, have Internet access, and be able to speak/read in English or Spanish.

### Procedures

Caregivers were enrolled in the study between July 2019 and February 2023. As part of the daily diary questionnaire and without any specific instruction encouraging caregivers to participate in an activity, caregivers were asked to report on whether they engaged in a shared activity with their loved one in the prior 24 hr. After baseline assessment, caregivers received a daily survey by email or by phone using the interactive voice response system for 21 days. Caregivers completed the daily survey sometime between 7 a.m. and 12 p.m. each day for the prior day, with reminders sent twice by email, phone, or text (caregivers’ preference). If they did not complete the survey during this time window, it was considered as missing. Caregivers received $40 for completing the baseline assessment and $2 per diary survey. Payments for completing the daily diary surveys were sent via an Amazon e-gift card to their email address, with the initial $5 sent after baseline and the remaining amount sent after the 21 days of diaries. All study participants signed an informed consent prior to start of the study. Study procedures were designated as exempt by the Institutional Review Board.

### Measures

Baseline demographic characteristics about the caregiver included age, sex, race, ethnicity, education, and relationship to the person living with dementia. The baseline demographic charactersitics of person living with dementia included their age and sex as reported by the caregiver. Health characteristics included caregiver depression measured by the Patient Health Questionnaire (PHQ-9; Kroenke et al., 2001). Individuals with PHQ total score ≥10 were considered as having moderate or severe depression. We used the Katz Index of Independence in Activities of Daily Living to assess person’s living with dementia limitations in their activities of daily living (Katz, 1983).

### Dependent Variable

#### Behavioral and psychological symptoms of dementia

Using daily diaries, caregivers were asked to indicate the number of times eight of individual BPSD occurred during the day and during the previous day (up to five times). These BPSD included restlessness, mood changes, resisting care, property destruction, disinhibition, aggressive verbal behaviors, aggressive physical behaviors, and any other bothersome behaviors. These selected BPSD were used in prior daily diary research (Fauth et al., 2006; Pickering et al., 2020).

#### Predictor—caregiver stress

Caregivers were asked the following question, “How stressed were you about the caregiving responsibilities for your relative that you had to do?” Response options ranged between 1 = “not at all stressed” to 5 = “very much stressed,” with 0 = “not applicable.”

#### Moderator—shared activity

Caregivers were asked the following question in the daily diary. “Did you do a pleasant noncare activity with your relative with dementia? Such as doing something together you both enjoy?” The caregiver responses were coded as “Yes” or “No.” The caregivers were not given specific examples of shared activities in the question prompt.

### Data Analysis

**Figure 1.**
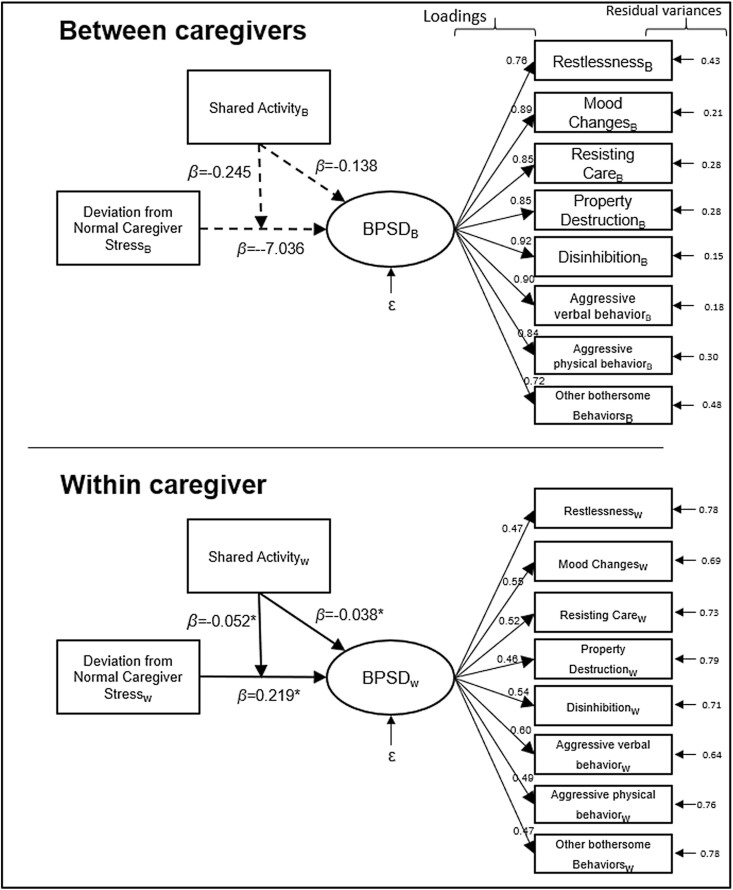
Path diagram of multilevel structural equation modeling. BPSD = behavioral and psychological symptoms of dementia.

We conducted a moderation model in a multilevel structural equation modeling (SEM) framework with daily observations clustered within individual caregivers (Figure 1; Hine et al., 2016; Matsueda & Press, 2012; B. Muthén & Asparouhov, 2011). Multilevel SEM allows to examine relationships between variables on multiple levels of observation including latent constructs. The BPSD had eight indicators, which were specified as a latent variable at the between-person level and the within-person level. A latent variable was used for modeling BPSD because it can help create a variable that is a function of the variance–covariance matrix of the eight observed BPSD indicators. Therefore, the common variance across the eight indicators is reflected via the latent variable. The predictor variable protective factor (i.e., whether a shared activity was done) was introduced as an observed binary/categorical variable (YES/NO) into the analysis. The caregiver stress variable was a Likert-type variable with five categories, and it was introduced as a person-mean centered observed variable for each individual caregiver to capture daily fluctuations around their typical levels of stress. To calculate the person-mean centered scores, a mean score for each participant across 21 days was calculated or all available days, then those means were subtracted from each participants’ diary scores. As a result, the person means was replaced by zero, and the values above the mean and the values below the mean were represented by positive and negative values, respectively. Maximum likelihood estimation algorithm with robust standard errors (*SE*s) was used to estimate the model parameters and the confidence intervals (CIs; B. Muthén, 2010). At the within-person level, we compared an individual’s daily experience to their own typical daily experience. At the between-person level we compared, on average, how these variables relate to one another across participants. The data set had two types of missing data: missing data on a given item on a given day and missing an entire day. The first type of missing data was minimal, and the second type of missing data was 18%. All the missing data were imputed using Mplus 8.8 (L. K. Muthén & Muthén, 2017) with the multilevel structure of the data taken into account. First, for the latent dependent variable BPSD, an empty (without any predictors) multilevel latent variable was run to see both the cross-level variability on the indicators and how well this measurement model fits the data. Then, the equation below, which shows the hypothesized multilevel moderation model that was run.

Level 1 equation:


$$\begin{array}{l} BPSD_{it}=\beta_{0t}+\beta_{1t}Stress_{it}+\beta_{2t}SA_{it}+\beta_{3t}Stress_{it}SA_{it}+\varepsilon_{it} \end{array}$$

Level 2 equation:


$$\begin{array}{l} BPSD\_between_{i}=\beta_{0}+\beta_{1}Stress_{i}+\beta_{2}SA_{i}+\beta_{3}Stress_{i}SA_{i}+\varepsilon_{i} \end{array}$$

The Level 1 equation above represents the within part of the model, which has random intercepts while Level 2 equation represents the between part of the model. In both equations, BPSD is a latent variable that has a scale of a standard normal distribution. SA stands for shared activity. The statistical significance cutoff was set at 0.05.

## Results

Our sample consisted of 453 caregivers (mean age 51.6, standard deviation [*SD*]: 14). Both caregivers and persons living with dementia were predominantly female and non-Hispanic White. Approximately 22.2% of caregivers were spouses or partners to persons living with dementia with 24% being below the poverty level (Table 1). The average number of diaries completed per caregiver was 17.18 (out of 21 days).

**Table 1.** Demographic Characteristics of Caregivers and Persons Living With Dementia

Variable	Mean ± *SD*	*N* (%)
Person with dementia (*n* = 453)		
Age in years (range: 56–100)	78.6 ± 9.1	
Female		265 (58.5)
Race		
White		302 (66.7)
Black		105 (23.2)
Other		46 (10.2)
Activities of daily living	3.9 ± 2.5	
Caregiver (*n* = 453)		
Age in years (range: 20–89)	51.6 ± 14.0	
Female		394 (87.4)
Race		
White		304 (67.1)
Black		101 (22.3)
Other		48 (10.6)
Relationship type		
Spouse/partner relationship type		101 (22.2)
Child		239 (52.8)
Grandchild		53 (11.7)
Nontraditional		46 (10.2)
Poverty level		
Above poverty level		344 (75.9)
Below poverty level		109 (24.1)
Hours care for others	5.1 ± 11.6	
Moderate or severe depression		144 (31.8)
Number of caregivers reporting doing a shared activity at least once		444 (98.0)

*Note*: *SD* = standard deviation.

The descriptive statistics (counts and proportions) for the indicators of BPSD latent variable, caregiver stress, and the shared activity were displayed in Table 2. The indicators of the BPSD had six categories each indicating the number of times the event occurred. After the variable was centered around its mean for the multilevel analysis, it had a mean of zero, and a *SD* of 0.94 with a range between −4.63 and 4.21. Further, findings showed that 444 of the 453 caregivers had at least one shared activity with a person living with dementia. Shared activity frequency showed that the caregivers and the persons living with dementia had a shared activity together for 57.37% of the days.

**Table 2.** Descriptive Statistics for the Behavioral and Psychological Symptoms of Dementia (BPSD), Caregiver Stress, and Shared Activity

BPSD indicators	0 times	1 time	2 times	3 times	4 times	5 times	NA	1	2	3	4	5	Yes	No
Restlessness	2,577 (27)	2,319 (25)	2,631 (28)	1,155 (12)	509 (5)	32 (3)								
Mood	3,675 (39)	2,205 (23)	1,990 (21)	896 (9)	413 (4)	334 (4)								
Resisting care	3,907 (41)	1,971 (20)	1,833 (19)	926 (10)	438 (5)	438 (5)								
Destruction	6,022 (63)	1,882 (20)	807 (9)	384 (4)	196 (2)	222 (2)								
Disinhibition	5,111 (54)	1,851 (20)	1,373 (14)	583 (6)	289 (3)	306 (3)								
Verbal	5,387 (57)	1,833 (19)	1,098 (11)	574 (6)	280 (3)	341 (4)								
Physical	6,251 (66)	1,888 (20)	677 (7)	305 (3)	157 (2)	235 (2)								
Any other	2,703 (28)	2,170 (23)	2,598 (27)	1,131 (12)	541 (6)	370 (4)								
Caregiver stress^a^	468 (5)	2,385 (25)	3,192 (33)	1,868 (20)	988 (10)	612 (7)	468 (5)	2,385 (25)	3,192 (33)	1,868 (20)	988 (10)	612 (7)		
Shared activity													4,458 (57)	3,311 (43)

*Notes*: All variables had less than 1% of item data missing; numbers outside and inside of a parenthesis represent number of times that category was selected and their counts (proportions), respectively.

^a^Value labels of caregiver stress: NA = not applicable, 1 = not at all stressed, 2 = slightly stressed, 3 = somewhat stressed, 4 = moderately stressed, 5 = very much stressed.

Two models were run: (i) a multilevel latent variable measurement model for BPSD with its indicators, and (ii) a multilevel structural equation model to test the moderation effect of shared activity on the relationship between caregiver stress and BPSD. The fit of the models to the data is displayed in Table 3 and both models fit to the data well. Root mean square error of approximation was below traditionally accepted levels of the model-data fit in SEM (Hu & Bentler, 1999). The standardized root mean square residual (SRMR) within and the SRMR between fit indices can be used to evaluate the fit for within and between levels, respectively. The SRMR between value is below the traditionally accepted threshold level of 0.08, which indicates a satisfactory level of model-data fit for both models.

**Table 3.** The Fit of the Models to the Data

Fit index	Measurement model	Moderation model
Number of parameters	40	45
Log-likelihood	−106,128.07	−130,606.133
Akaike Information Criterion	212,336.148	261,302.265
Bayesian Information Criterion	212,622.565	261,624.484
Comparative fit index	0.945	0.934
Tucker–Lewis index	0.923	0.917
Root mean square error of approximation	0.031	0.027
SRMR between	0.046	0.065
SRMR within	0.031	0.03

*Note*: SRMR = standardized root mean square residual.

Table 4 displays the intraclass correlation coefficients for the BPSD indicator variables, which indicate that there is substantial amount of variance at each level for each indicator. Within-level standardized loadings and the between-level standardized loadings are displayed on the same table. All loadings were statistically significant, and they are large enough to argue that BPSD latent variable had strong relation with its indicators at each specific level.

**Table 4.** Item ICC and Item-Factor Loadings Based on Standardized Model Parameters

Behavioral and psychological symptoms of dementia	ICC	Lambda_within	Lambda_between
Restlessness	0.384	0.468	0.757
Mood	0.375	0.554	0.889
Resisting care	0.404	0.52	0.848
Destruction	0.459	0.456	0.851
Disinhibition	0.402	0.536	0.924
Verbal	0.41	0.599	0.903
Physical	0.472	0.494	0.836
Any other	0.36	0.473	0.723

*Note*: ICC = Intraclass correlations.

At the within-person level, when caregiver’s stress was higher than their typical average stress level, there was a significant increase in the amount of BPSD that day by a unit of 0.219 (estimate 0.219, *SE* = 0.02, 95% CI: 0.179, 0.259, *p* value <.001). On days when the caregivers engaged in a shared activity together with person living with dementia, there was a significant decrease in the BPSD that day (estimate −0.038, *SE* = 0.016, 95% CI: −0.069, −0.007, *p* value = .018). Furthermore, the effects of engaging in a shared activity also decreased the impact of caregiver stress by 0.052 (estimate −0.052, *SE* = 0.018, 95% CI: −0.087, −0.017, *p* value = .004). At the between-person level, we found no relationship between persons living with dementia who engaged in shared activities and BPSD. The moderation model explained 14.3% and 8.6% of the variability in BPSD at within level and the between level, respectively (Table 5). Unstandardized parameter estimates and corresponding 95% CIs are provided in Supplementary Table 1.

**Table 5.** Unstandardized Parameter Estimates and 95% CIs

Variable	Estimate	*SE*	95% CI		*p* Value
Within level					
Moderating effect of shared activity	−0.052	0.018	−0.087	−0.017	.004
Effect of stress on BPSD	0.219	0.02	0.179	0.259	<.001
Effect of shared activity on BPSD	−0.038	0.016	−0.069	−0.007	.018
Between level					
Moderating effect of shared activity	0.245	10.96	−21.726	21.236	.982
Effect of stress on BPSD	−7.036	61.083	−126.76	112.687	.908
Effect of shared activity on BPSD	−0.138	1.19	−2.47	2.194	.908

*Notes*: BPSD = behavioral and psychological symptoms of dementia; CI = confidence interval; *SE* = standard error.

## Discussion and Implications

The purpose of the present work was to examine the moderating effect of shared activities between a person living with dementia and their caregiver on the association between the number of BPSD and caregiver stress. On the within-level we found that when the dyad engaged in a shared activity, persons living with dementia experienced fewer BPSD. In addition, engaging in a shared activity decreased the impact of caregiver stress on BPSD in persons living with dementia. We found no differences in the amount of BPSD across caregivers who engage or did not engage in a shred activity. Therefore, Hypothesis 1 and 2 were supported. The results of our study indicate that doing a shared activity is associated with reduced BPSD among persons living with dementia and may buffer the impact of caregiver stress on BPSD.

Nonpharmacological approaches have been historically encouraged for managing BPSD (Bessey & Walaszek, 2019). The management plan for BPSD is highly individualized and based on various factors, such as patients’ personal characteristics, resources, availability of caregivers, and the context in which BPSD occurs (Bessey & Walaszek, 2019). The management plan may include tailored activities. Sideman et al. (2023) indicated that shared activities between persons living with dementia and their caregivers are opportunities for both dyad members to improve their connection experience and its positive outcomes, such as improved health and well-being. Our findings highlight the importance of care interventions to support shared activities as effective methods for managing BPSD beyond the delivery of caregiving tasks.

Caregiver distress is often treated as an outcome worthy of intervention (Butler et al., 2020; Cheng & Zhang, 2020) and unaddressed/unmanaged caregiver distress may cause harm to the person living with dementia and increase BPSD (Pickering et al., 2022). In the current study, shared activity moderates the relationship between distress and BPSD, which highlights a potential target for future interventions. The evidence for care interventions that focuses on managing BPSD and improving caregiver well-being remains limited (Butler et al., 2020). Caregiver interventions should focus on effective approaches in relieving caregiver distress, although completely relieving all distress is unlikely. Instead, it may be a preferable strategy to relieve distress whenever possible and emphasize self-management and coping strategies (Basu et al., 2015; Lorig et al., 2012, 2019), which may reduce the impact of distress associated with caregiving. Strategies that incorporate shared activities into daily life (like listening to music during mealtimes or bathing) may be more effective and scalable than prescribed activities because these shared activities address the unmet needs of a person living with dementia while also helping caregiver manage their stress.

As with any research, this study had several limitations. The participants were recruited from a convenience sample, which might limit the generalizability of the findings due to possible selection bias. Future studies should attempt to collect more information about the person living with dementia to better contextualize the BPSD and caregiver stress (and their potential sources). While our study asked caregivers to indicate in the diaries whether they had shared activities (yes/no) with care recipients, we did not ask caregivers to describe the nature of the shared activity because interventions that promote shared activity as a BPSD strategy emphasize personalized activities, which was the hypothesis we were testing. Because the hypothesis was supported, future research is recommended to investigate the nuances and heterogeneity of shared activities reported by caregivers to understand the complex relations between shared activities and caregiver stress as well as BPSD and how relationship type may affect this. Although the daily diary method allows researchers to examine participants’ behaviors repeatedly over time with few recollection errors and retrospective bias (Bolger et al., 2003), the diary entries are still reliant on participant’s perceptions of their behaviors and lived experiences, which could be prone to social desirability bias. Future studies are recommended to complement the daily diary method with other data collection methods such as observations and ecological momentary assessments to capture real-time data on caregivers’ and care recipients’ behaviors. Also, innovative and tailored research methods are needed to engage and solicit perspectives from care recipients or persons living with dementia in studies related to their dyadic interactions with caregivers.

The strengths of the study included a large and diverse sample of community-dwelling coresiding caregivers for persons living with dementia to allow for robust analysis of daily shared activities, BPSD, and caregiver stress. The sample was diverse in terms of race/ethnicity, with half the sample representing persons from minoritized groups. This design allowed for examining the protective effects of shared activities on caregiver stress and BPSD as they occurred in the context of daily life enhancing the ecologically validity of the findings. Furthermore, tracking BPSD and caregiver stress using daily diaries may have reduced recall bias and measurement issues that occur with aggregate group-level estimates of BPSD.

Our study has significant implications for future research directions. Shared activities should be considered a key intervention component for dementia caregivers; it has both a protective momentary effect (reducing BPSD and stress on the day the activity happens) and a cumulative protective effect (people who do shared activities overall have lowered BPSD). Future research is needed to explore which shared activity interventions may be better at creating opportunities for meaningful/intimate connection than others and how engagement in shared activities may change over time. Based on our study, we believe that additional high-quality studies are needed to assess nonpharmacological activities to manage BPSD in persons living with dementia and caregiver stress, especially in relation to different cultural groups and ethnicities. Future research is needed to optimize the dosing and duration of shared activities to gain the cumulative protective effect and identify barriers preventing caregivers from engaging in shared activities with their care recipient.

In summary, in this study we examined shared activities as a protective factor against BPSD among coresiding caregivers and persons living with dementia. We found that as caregiver’s stress levels increased, persons living with dementia experienced a higher number of BPSD. When caregivers reported engaging in a shared activity, they experienced less stress and there was a significant reduction in BPSD on the same day. Taken together, our findings contribute to the knowledge gap regarding factors associated with BPSD. In addition, our study findings demonstrate a possible mechanism that can be further explored in nonpharmacological interventions guided by the National Institutes of Health stage and behavioral change models (Nielsen et al., 2018; Onken et al., 2013). Given the growing number of caregivers in the United States, it is essential to identify factors that may lessen caregiver stress and BPSD in persons living with dementia.

## Supplementary Material

igae034_suppl_Supplementary_Table

## Data Availability

Data are available upon request of the corresponding author for the purposes of replicating the findings. This study was not preregistered.
