# Supplementary material for: Shared Activities as a Protective Factor Against Behavioral and Psychological Symptoms of Dementia and Caregiver Stress
Source: Innov Aging. 2024 Mar 11;8(5):igae034. doi: 10.1093/geroni/igae034 (PMC11037273; doi:10.1093/geroni/igae034)
Supplement: igae034_suppl_Supplementary_Table [file igae034_suppl_supplementary_table.docx]

***Innovation in Aging* Supplementary Material: Petrovsky et al. Shared Activities as a Protective Factor Against Behavioral and Psychological Symptoms of Dementia and Caregiver Stress.**

**Supplementary Table 1. Standardized parameter estimates and 95% confidence intervals**

|  | Estimate | SE | 95% CI | | P value |
| --- | --- | --- | --- | --- | --- |
| *Within level* |  |  |  |  |  |
| Moderating effect of shared activity | -0.073 | 0.025 | -0.122 | -0.024 | 0.003 |
| Effect of stress on BPSD | 0.424 | 0.029 | 0.367 | 0.481 | <0.001 |
| Effect of shared activity on BPSD | -0.033 | 0.014 | -0.06 | -0.006 | 0.017 |
|  | R_squared | 0.143 |  |  |  |
| *Between level* | | | | | |
| Moderating effect of shared activity | 0.029 | 1.291 | -2.559 | 2.094 | 0.982 |
| Effect of stress on BPSD | -0.198 | 1.719 | -3.567 | 2.631 | 0.908 |
| Effect of shared activity on BPSD | -0.057 | 0.492 | -1.021 | 0.752 | 0.908 |
|  | R_squared | 0.086 |  |  |  |

Note: BPSD = behavioral and psychological symptoms of dementia; SE = standard error, CI = Confidence interval
